# North Pacific Baleen Whales as a Potential Source of Persistent Organic Pollutants (POPs) in the Diet of the Indigenous Peoples of the Eastern Arctic Coasts

**DOI:** 10.3390/toxics7040065

**Published:** 2019-12-17

**Authors:** Pavel Chukmasov, Andrey Aksenov, Tatiana Sorokina, Yulia Varakina, Nikita Sobolev, Evert Nieboer

**Affiliations:** 1 Arctic Biomonitoring Laboratory, Northern (Arctic) Federal University named after M. V. Lomonosov, Severnaya Dvina Emb. 17, Arkhangelsk 163002, Russia; a.s.aksenov@narfu.ru (A.A.); t.sorokina@narfu.ru (T.S.); yu.andreeva@narfu.ru (Y.V.); megabait8@gmail.com (N.S.); 2 Department of Biochemistry and Biomedical Sciences, McMaster University, Hamilton, ON L8S 4L8, Canada; nieboere@mcmaster.ca

**Keywords:** gray whales, bowhead whales, North Pacific, POPs, indigenous people’s nutrition

## Abstract

Among marine mammals, gray and bowhead whales contain large amounts of fat and thereby constitute crucial dietary components of the traditional diet of indigenous peoples of the Eastern Arctic. Despite the high nutritional and cultural value of gray and bowhead whales, there is a risk of persistent organic pollutant (POP) intake by indigenous individuals who use marine mammals as their main source of fat. POPs are lipophilic pollutants and are known to accumulate and magnify along the marine food web. Consumption of foods contaminated by POPs can perturb the endocrine, reproductive, and immune systems, and can potentially cause cancer. Moderate to relatively high concentrations of POPs have indeed been reported in the edible tissues of gray and bowhead whales consumed by indigenous peoples of the North Pacific Ocean. Even though their consumption is potentially harmful, there is no regular monitoring of eco-toxicants in the foods consumed by the indigenous peoples of the Eastern Arctic. In our view, the routine analyses of consumable parts of whales and of comparable nutritional items need to be included in the Russian Arctic Biomonitoring Programme.

## 1. Introduction

Human life in all coastal settlements is inextricably linked with the sea, and often involves fishing, collecting molluscs and algae, and the hunting of marine mammals. The harsh arctic climate and lack of a variety of food sources and of vitamins and minerals have motivated some indigenous communities of the north to live near the sea in order to hunt marine mammals (i.e., seals, walruses and cetaceans). These activities have existed for thousands of years and continue to ensure the survival of peoples living in the Bering Strait region [1].

Marine mammals contain a large amount of fat and led to the adoption of a protein-fat diet by the indigenous peoples of the Arctic. This arctic diet has been embraced for thousands of years and is suitable for human survival and adaptation in harsh environments. Currently, hunting of marine mammals exists in almost all countries of the Arctic region, namely: the Russian Federation (Chukotka), the USA (Alaska), Canada, Norway, Iceland and Denmark (more specifically, Greenland and the Faroe Islands). In contrast to the available market foods, nutritional studies have shown that high-fat traditional foods consumed in arctic indigenous communities contain high amounts of nutrients, protein and polyunsaturated fats. In this context, the replacement of the latter by the former has been linked to increases in obesity and chronic diseases in arctic indigenous communities [1,2,3,4].

Traditional whaling is an integral part of the existence of some indigenous inhabitants of the Arctic region, both in terms of physical survival and cultural continuity that ultimately determine the unique nature and character of the peoples of the Far North. The main commercial species in Chukotka is the gray whale, while in Alaska, it is the bowhead whale. The annual catch quota for gray and bowhead whales for the indigenous inhabitants of the Chukotka is 140 animals (120 in 2017) and 67 in Alaska [5]. Catches are regulated by the International Whaling Commission (IWC) [5]. Its objective for the management of aboriginal subsistence whaling is to ensure that hunted whale populations are maintained at (or brought back to) healthy numbers, thereby enabling indigenous communities to hunt at levels appropriate for meeting their cultural and nutritional needs.

Whale tissues (i.e., blubber, muscles, liver and skin) are considered healthy foods for harsh arctic climate conditions [6,7]. However, their regular consumption may incur the intake of high levels of toxic lipid-soluble contaminants. For example, the intake of lipophilic persistent organic pollutants (POPs) could be at toxic levels in some whale species due to the bioaccumulation and biomagnification of these toxicants. Indeed, several studies have shown that individuals who consume a large amount of seafood have a high POP body content [8,9], including polychlorinated dibenzo-p-dioxins (PCDD), dibenzofurans (PCDF) and polychlorinated biphenyls (PCBs). In this context, special attention has focused on indigenous peoples consuming large amounts of seafood, including marine mammal fats [8,9]. Dewailly et al. [8] observed rather high concentrations of POPs in the blood of Greenlandic Inuits who regularly consumed traditional high-fat marine foods when compared to individuals living in industrialized regions. Health effects associated with pollutants do not appear immediately after ingestion but often do so several years after exposure [10]. Breast milk has also been shown to be a potential source of POPs for newborns [11].

In the subpolar Arctic region, there has been minimal direct use of POP chemicals, namely chlorinated, brominated, and/or fluorinated organohalogen compounds (OHCs). Nevertheless, POPs (or pertinent precursors), their metabolites and decomposition products are transported to the Arctic from more southern latitudes by way of long-range atmospheric transport and, of course, ocean currents and rivers [12,13,14]. For arctic wildlife, it should also be noted that there are numerous natural (environmental and physiological) and anthropogenic factors that can affect exposure to pollutants, including climate change, invasive species and pathogens, changes in the dynamics of the food web and predator-prey interactions [15,16,17,18].

Over the past two decades, the concentrations of many of these pollutants in marine biota have decreased, although the concentrations of PCBs and chlordanes in wildlife tissues have remained relatively high [19]. Nevertheless, thousands of new synthetic chemicals are produced every year, and there have been recent reports of the presence of brominated flame retardants (BFRs) and polybrominated diphenyl ethers (PBDEs) [14] in humans and marine biota [20,21]. Specifically, there is a lack of relevant data on the concentrations of POPs in the tissues of bowhead and gray whales, which are important components of the traditional foods consumed by the indigenous peoples of Alaska and Chukotka [10]. In the current article, we summarise the available data on the contents of POPs in gray and bowhead whale tissues consumed by them.

## 2. Survey Findings and Discussion

### 2.1. Geography

**Figure 1:**
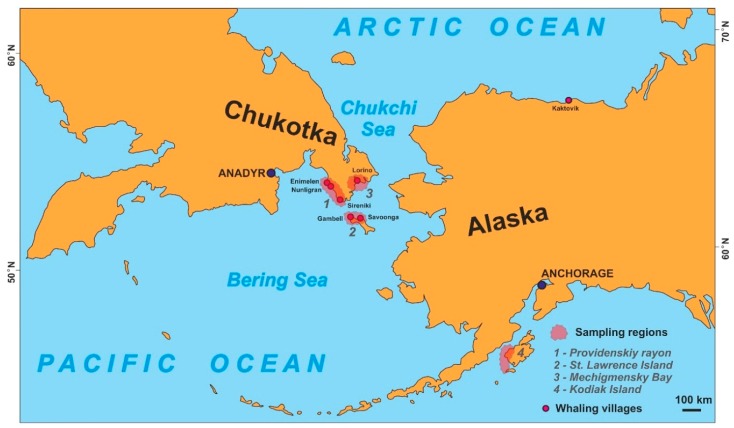
The main whaling villages in Chukotka and Alaska (red points).

Our primary geographical focus in this article are the Russian coastal regions of Chukotka and Alaska, which are located in the northern part of the Pacific Ocean (see Figure 1). According to the recent Russian populational census, the indigenous population of Chukotka constitutes approximately 33% of its total [22]. By comparison, about 15% of the Alaskan population identify themselves as indigenous [23]. These two populations live on different continents and in different countries and geographically are separated by the narrow Bering Gulf, as depicted in Figure 1. Not surprisingly, there has been a historical connection. Both populations live in a harsh sub-arctic/arctic climate that is characterized by relatively low annual temperatures, long and cold winters, as well as short and wet summers; they also endure high average annual winds and frequent snowstorms and hurricanes [24]. In addition, the infrastructure is deemed to be poor, as it features low nutritional diversity and a lack of availability of plant-based foods, as well as ongoing social issues [5,25]. Furthermore, climate change has contributed to negative socioeconomic trends in relation to permafrost melting, soil erosion and shorter duration of viable ice routs [24]. In this context, the hunting and consumption of marine mammals have not diminished, and has not been replaced by western nutrition. Whaling generally takes place in remote villages on the shores of the Northern component of the Bering Sea shore (see Figure 1) that are along the migration routes of gray and bowhead whales [5].

### 2.2. Consumption of Whales and Its Influence on Human Health

In whaling communities, whale meat, fat and blubber are distributed on the basis of need. All inedible parts, such as bones and baleen, are used as raw material for making toys, tools, decorations, etc. Questionnaire surveys [26] have shown that 86% of the Chukotka region’s coastal indigenous population indicated whaling as a main source of food. The annual consumption of whale meat in 2000 was 52 kg per person [26]. Of those interviewed in the villages Enmelen, Nunligran and Sireniki, 26% indicated that the gray whale was the main source of fat [25,26]. In all, the average consumption by Alaskan indigenous people of bowhead whale products has been estimated at 499,000–907,000 kg per year [5]. Their high fat and protein contents appear to decrease the risks of obesity and cardiovascular diseases [27]. Moreover, the high levels of omega-3 fatty acids in these products appear to normalize glucose metabolism [28]. This suggests that the consumption of whale products provides important health and nutritional benefits [6]. Despite the high nutritional quality and cultural value of gray and bowhead whales, their consumption entails the potential intake of POPs present in whale tissue fat.

Studies of cetaceans and other mammals, including humans, have shown that POPs can negatively affect the endocrine, reproductive and immune systems and potentially are carcinogenic [5]. It has been shown that during the 1961–1990 period the indigenous coastal Chukchi and Inuit living in Chukotka (Russia) had higher cancer mortality rates than the Russian population. Relative to the age-standard cancer mortality risk in Russia, the overall risk was two-fold higher among men and 3.5-fold higher for women. Notably, high mortalities from esophageal and lung cancers were evident for the indigenous population of coastal Chukotka. Interestingly, the latter outcomes are comparable to those reported for other indigenous peoples of the Russian Arctic [29]. Similarly, the incidence of colorectal cancer is currently higher among the Alaskan Inuit than in Caucasians living in mainland USA [30]. Cancer is now the leading cause of death among Alaska Native peoples, and cancer mortality rates in Alaska are also significantly higher than in mainland USA [31]. Based on epidemiological studies, around 80% of all cancers are suspected to be related to environmental factors that include contaminant exposure and lifestyle [19].

The hazards of POPs to the ecosystem are related to their resistance to biodegradation and the potential for long-range transport [19,32]. Bioaccumulation and biomagnification of POPs and pesticides in food webs can potentially lead to their consumption by humans at toxic levels, and thereby impact public health [19,33]. Potential negative outcomes in both animals and humans can involve disorders of the liver, the central nervous, cardiovascular, endocrine and reproductive systems [34,35,36,37], as well as mutagenic/carcinogenic outcomes [6,38]. Furthermore, neurophysiological effects such as attention-deficit hyperactivity disorder, learning disorders and behavioural problems are suspected, including increased aggressiveness and poor coordination [37,39].

Due to the fact that POPs have negative impacts, bioaccumulate, biomagnify and are transported over long distances by air, sea currents and migratory species, their production and use are regulated internationally. The Stockholm Convention on Persistent Organic Pollutants was completed on 23 May 2001, and entered into force on 17 May 2004. Signatories to the Convention pledged to prohibit the production and use (with a few exceptions) of twelve distinct chemicals. The use of dichlorodiphenyltrichloroethane (DDT) was limited to the control of malaria and the unintentional production of dioxins and furans was curbed. In 2009, nine additional organic compounds were added. As of 26 September 2019, the number of signatories reached 183 [40]. An analysis of the implementation of the Stockholm Convention in terms of promoting monitoring and research was published recently [41]. It involved the Russian Federation as a case study.

### 2.3. Observed Concentrations of POPs in Whale Tissues

As a result of the transfer of POPs from southern latitudes to the Arctic, they bioaccumulate and biomagnify along the marine food web [11]. The extent of these processes depends on location and so does the accumulation of POPs in whales.

The gray whale feeds exclusively on benthic organisms. This unique food source often leads to the ingestion of sediment and other bottom materials. Consequently, exposure to pollutants associated with precipitation is possible for gray whales that in coastal areas feed on sediment and benthic invertebrates contaminated with anthropogenic compounds [42]. By contrast, bowhead whales feed exclusively on plankton and euphausiids, and filter water through the mouth [43].

One of the first studies of POPs in gray whales by Wolman and Wilson showed that dichlorodiphenyltrichloroethane-related compounds [i.e., DDT, dichlorodiphenyldichloroethane (DDD) and dichlorodiphenyldichloroethylene (DDE)] concentrations in the blubber of 6 out of 23 gray whales varied from 22 to 360 ng/g *w/w*, while the liver did not contain any of these compounds [44].

In subsequent studies of gray whales [42,43,45,46,47,48], concentrations of ƩPCB, ƩDDT, cis-nonachlor, trans-nonachlor, cis-chlordane, trans-chlordane (ƩCHLOR), hexachlorobenzene (HCB), hexachlorocyclohexane-isomers (ƩHCH) and dieldrin were quantified in blubber, liver, stomach contents, brain, muscle, kidney and mantak. The sampling was carried out in two locations, namely: Provdenskiy Rayon (Chukotka, Russia) and Kodiak Island (Alaska, USA). In addition, the bowhead whale was sampled at St. Lawrence Island (USA) (see Figure 1). For comparison, concentrations are also presented of selected POPs in gray whales from California and San-Francisco [42,45], as well as bowhead whale from the Northern part of Alaska (Kaktovik, see Figure 1), which could be a migrating part of the same population. The average amount (in ng/g, *w/w*) of organochlorine (OC) pollutants reported in gray and bowhead whale organs and meats are summarized in Table 1.

In a 1994 study, Varanasi et al. [42] examined tissue samples of migratory gray whales that were stranded from 1988 to 1991 across the entire west coast of the United States, from Alaska to California. POPs were detected in all samples examined. For example, the concentrations of PCBs and ∑DDEs in blubber ranged from 120 to 10 000 and from 11 to 2100 ng/g *w/w*, respectively. ∑PCB concentrations were higher than other POPs, including DDT and its derivatives DDE and DDD, both in blubber and liver tissue and independent of where the sample was taken. By comparison, the total concentrations of DDT and its derivatives were generally higher than other chlorinated pesticides such as chlordanes. Statistical analyses were somewhat limited by the availability of samples; for example, fat was available from all 22 animals, but the liver was collected from only 10 animals; in addition, the sex of two whales was unknown [42].

In another study [45], tissue samples were taken from 22 young gray whales harvested from Arctic feeding grounds located in a relatively anthropogenically untouched area of the western Bering Sea. The summed concentrations of ∑PCBs and ∑DDTs OCs in blubber ranged from 110 to 1300 and from 30 to 540 ng/g *w/w*, respectively [45]. By comparison, young whales cast ashore (i.e., stranded; also see [42]) contained significantly higher average concentrations of OCs than did young animals taken from feeding places. Presumably, this observation reflects the mobilization of OCs from adipose tissue, such as during migration and residence at breeding grounds [45].

Studies have shown that POPs are also present in the liver and muscles of gray whales consumed by the indigenous peoples of Chukotka [46,47]. More recent studies conducted using samples obtained during the annual indigenous whaling activities in Chukotka show that the total concentrations of OCs in the muscles and liver of gray whales varied from 297 to 3581 and 769 to 13 808 ng/g of lipids, respectively; and of HCB in whale blubber and mantak of 180–200 ng/g *w/w*) [46,47].

Hoekstra et al. in 2002 showed that POPs are also present in bowhead whales [49]. They analyzed 72 fat samples and 23 liver samples from bowhead whales harvested during the traditional whaling periods from 1997 to 2000 in Barrow (now Utkigvik), Alaska. The rank order of OC group concentrations (geometric mean, *w/w*) in bowhead blubber samples were: toxaphene (455 ng/g) > ∑PCBs (410 ng/g) > ∑DDT (331 ng/g) ≥ ∑HCHs (203 ng/g) ≥ chlordanes and related isomers (∑CHLOR; 183 ng/g) > chlorobenzenes (∑CIBz; 106 ng/g). In liver, ∑HCH (9.5 ng/g; wet weight) was the most abundant ∑OC group, followed by ∑PCBs (9.1 ng/g) ≥ TOX (8.8 ng/g) > ∑CHLOR (5.5 ng/g) > ∑CIBz (4.2 ng/g) and ≥ DDT (3.7 ng/g). The dominant analyte in blubber and liver was p,p-DDE and α-HCH, respectively [49]. In a subsequent report by Hoekstra et al. in 2005, samples of blubber, mantak and other tissues of 5 bowhead whales were taken during the traditional whaling activities of 1997–1999, again in Barrow [49]. OC contaminants were detected in all samples analyzed.

It is notable that the concentration of some POPs (e.g., 3200 ng/g ƩPCB, see Table 1) and, based on the food consumption rates, according to Dudarev et al. [25], could exceed the World Health Organization (WHO) tolerable level of intake [50]. Together with the intake of other POPs, this is worrying and unacceptable. In general, the concentration ratios for the POPs in gray and bowhead whales followed the concentration sequence ƩPCB > ƩDDT > ƩCHLOR = HCB. Moreover, most of the ƩPCB concentrations found in the blubber of whales exceeded the maximum levels allowed in marine oils by the European Union (EU) Commission regulations [51], namely 200 ng/g fat. Note that these limits were developed for marine fish and fish oil, and not for meat and organs of marine mammals. In any case, most of the samples of whale blubber and liver exceeded the estimated maximum limits and their consumption at an annual intake level of 49 kg of marine mammals (including whales) would exceed the WHO tolerable level intake 20 ng/kg body weight per day for mixtures of PCBs, and could even approach the minimum level that caused adverse effects in animals (5000 ng/kg body weight per day) [50].

Generally speaking, high variations in the concentrations of PCBs, DDT, HCB and other POPs were observed and depended on the part of the whale analyzed and varied from 10 ng/g to 6 g/g for the sum of POPs. Clearly, the maximum concentrations of POPs in whales occurred in blubber and liver and they constitute significant components of the indigenous nutritional intake. The ΣPCBs concentration was higher for gray whales in comparison to Bowhead whales. Another organ that has high concentrations of POPs is the liver. For muscle tissue, which is also one of the main sources of protein for indigenous individuals, the average content of POPs was more than 10 times lower than in blubber and liver. However, there is still a lack of information about the concentration of POPs in gray whales, which should, therefore, be included in any pan-Russian biomonitoring program.

In general, the concentrations of POPs in the bodies of young cetaceans do not differ. But after reaching puberty, differences appear. Higher POPs are present in the bodies of mature males and cows that feed on their mother’s milk, suggesting the transfer of OCs from females to their young during pregnancy and feeding [43,52,53].

Based on the analytical data reviewed above, it can be concluded that POPs are present in gray and bowhead whale meats/tissues consumed regularly by the indigenous people of Chukotka and Alaska. Consequently, there is no doubt that their primary food source has been and is contaminated and constitutes a major source of POPs. Unfortunately, there is not enough data for extensive statistical analyses.

### 2.4. Pertinent Issues

Climate change in the Arctic is contributing to the possibility of the economic development of isolated regions previously inaccessible for this purpose. However, experience has shown that economic activity often introduces environmental pollution issues pertaining to both land and sea [54].

Some animal species may well develop new habitats and feeding habitats in response to climate change [55], including those that are especially susceptible to anthropogenic pollution. Gray whales make the longest seasonal migrations among mammals. During a year, these animals swim 12–19 thousand km [56], and because of climate change may establish new feeding grounds [55]. For example, individual gray whales have been observed uncharacteristically in the Mediterranean Sea, off the coast of Namibia and inland in the Laptev Sea [55,57,58].

**Figure 2:**
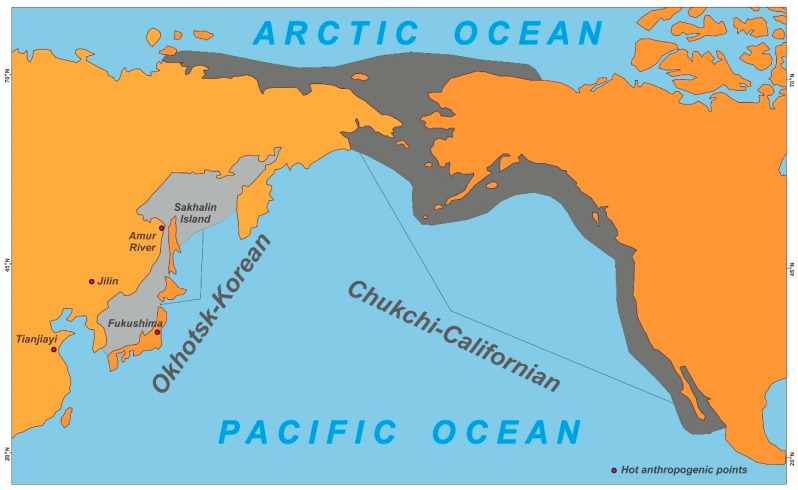
The map with migration paths of two gray whale populations.

There are two gray whale populations, namely: Okhotsk-Korean and Chukchi-California (Figure 2). The first of these appears to winter and probably multiply near the coast of Korea or Japan, while in summer, they feed in the Sea of Okhotsk on the northeast shelf of Sakhalin. These whales were almost exterminated as a result of commercial whaling and, based on approximate calculations; their current number is estimated to be around 200. The second population overwinters and multiplies in the Gulf of California (Mexico) and in the summer feeds in the Chukchi and Bering seas and the Beaufort Sea. Furthermore, they rarely enter the East Siberian Sea. They are more numerous, numbering approximately 26,000. The international quota allocated for annual aboriginal whaling is based on this estimate [59].

Although the western population of gray whales is considered to be independent, it is possible that they can mix and interbreed with the eastern group. Satellite tagging, photo-identification and genetic analysis have documented a movement of gray whales between the Western and Eastern Pacific Oceans [59,60,61]. It would be interesting to compare the presence of environmental contaminants in these two populations. As indicated earlier, the eastern group feeds off the coast of Chukotka (see Figure 2) and is hunted by indigenous people. By comparison, the western population feeds off the coast of northeastern Sakhalin. The latter group may well be more susceptible to the accumulation of POPs than the Eastern population because it is more sensitive to anthropogenic influences than the Chukotka feeding area. This is due to several factors: (i) the Amur River flows into the Sea of Okhotsk, and its output reaches the feeding grounds of the gray whales in Piltun Bay in northeast Sakhalin; (ii) there is strong anthropogenic pollution of the Amur waters; and (iii) agricultural activities have created serious environmental threats in the basin [62]. Although there are no specific statistics or calculations on how much of the chemical fertilizers or pesticides used in agricultural activities end up in the environment, their excessive use has caused serious pollution of surface waters in the Amur River basin [63,64,65]. Intensive shipping, high population density, close proximity to industrial exploration sites and past man-made disasters increase the risk of various toxic wastes getting into the Sea of Okhotsk. Of the more recent major industrial catastrophes in this area, the 2005 accident at a chemical plant in the city of Jilin (China) stands out. It resulted in 100 tons of chemicals, including benzene, phenylamine and nitrobenzene being released into the Songhua River, and subsequently into the Amur River. Other examples are the Fukushima nuclear accident in Japan in 2011 and an explosion in China at the Tianjiayi chemical plant in 2019; the latter specialized in the production of pesticides [66,67,68].

If the two populations of gray whales are closely related, it is possible that they feed both off the coasts of Sakhalin Island and Chukotka, where they can be harvested during the aboriginal whaling season. Providing that whales feeding off the coast of Sakhalin Island contain more toxic substances, the risk of intake by indigenous populations is thereby enhanced. In this context, official information from the International Whaling Commission on the so-called “stinking” whales is pertinent. The meat and fat of some gray whales have been known to have a strong chemical smell, which made them inedible. The reason for this phenomenon remains unclear, despite investigation [5]. It seems possible that this could be due to the nutritional characteristics of benthic organisms and the intake of toxic substances along with sludge and sediments.

Clearly, more research is needed. To understand the origin and accumulation of toxic chemicals in whales better, it is necessary to obtain multiple samples of animal fat from two feeding places. Since whaling is not conducted off the coast of Sakhalin Island, it seems possible to obtain samples of fat directly from healthy whales using the biopsy method, as well as from the occasional findings of dead whales along coastal areas.

## 3. Conclusions and Recommendations

### 3.1. Concluding Remarks

Protein-fat diets are necessary when living in a harsh climate. They are of high nutritional value and provide a source of essential fatty acids in the absence of the availability of other foods. Clearly, for coastal aboriginal communities in the Arctic, whaling provides a crucial food source.

It is clear from our review that POPs, especially ƩPCB, consumed by the indigenous peoples of the North Pacific are present in blubber and liver of gray and bowhead whales at levels that substantially exceed the EU limits for consumption. It is notable that the intake of ƩPCB could also exceed the WHO tolerable level intake of 20 ng/kg body weight per day when, on average, 49 kg of marine mammal tissues is consumed annually. Changing the diet to avoid this source of contamination poses a potential but unknown hazard. It potentially exposes coastal indigenous people to the risk of serious health issues associated with a lack of protein, retinol, certain fatty acids, as well as iron and zinc. The Government of Canada’s Canadian Arctic Contaminants Assessment Report (CACAR) and the Arctic Monitoring and Assessment Programme (AMAP) reports recommend that indigenous people continue to consume traditional foods because their nutritional value outweighs the potential risks associated with chemical contamination of their primary food source [69,70]. However, a reduction of the consumption of blubber and liver of whales could decrease the risk of intoxication by POPs. Furthermore, research on the content of POPs in the tissues of marine mammals will allow their identification and tracking. This should include new toxic compounds known to be present in the environment. It is important to focus on how they are transmitted through trophic links and their potential impact on human health. As indicated earlier, the presence of POPs in marine mammal fat tissues can be achieved by biopsy sampling without harming the animal. Biopsy sampling can also be used to track the presence of contaminants in marine mammals in areas other than their habitats where there is no whaling. Regular sampling by members of indigenous communities and subsequent analyses of gray whale fat would allow this species to be included in any ongoing Arctic biomonitoring program conducted in the Russian Federation. Such systematic monitoring would raise public awareness of potential health risks associated with the consumption of marine mammal tissues that are contaminated by POPs. Other marine mammals consumed by the indigenous people of the Arctic within the Russian Federation territory include bowhead whales (*Balaena mysticetus*), beluga whales (*Delphinapterus leucas*), walrus (*Odobenus rosmarus*), and some species of phocids.

### 3.2. Recommendations

To reduce the possible risks to unborn children, and those ingesting POPs from their mothers’ milk, it is suggested that the consumption of traditional foods be reduced by women during pregnancy and the lactation period.

To this end, it is recommended to provide pregnant and lactating women in remote villages with alternative food products to ensure the intake of essential nutrients that include vitamins and essential elements.

## Figures and Tables

**Table 1:** Summary of POPs concentrations in North Pacific gray and bowhead whales in ng/g (*w/w*) ^1^.

Tissue	ƩPCB	ƩDDT	ƩCHLOR	HCB	ƩHCH	Dieldrin	Ref.
**Gray whale**							
**Blubber**							
*n* = 17	630	150	140	230	- ^2^	-	[45]
*n* = 1	92	72	53	184	89	-	[47]
*n* = 5	1800	529	270	330	-	160	[42]
*n* = 4	1200	308	190	290	-	89	[42]
*n* = 4	3200	1000	650	850	-	450	[42]
*n* = 2	680	167	72	47	-	32	[42]
*n* = 4	780	217	110	130	-	52	[42]
*n* = 1	55	6.8	4	14	-	5	[42]
**Liver**							
*n* = 14	22	3	5	24	-	-	[45]
*n* = 7	-	1707 ^3^	-	-	3081 ^3^	-	[46]
*n* = 4	630	177	88	190	-	63	[42]
*n* = 2	940	184	100	200	-	64	[42]
*n* = 1	370	98	61	110	-	40	[42]
*n* = 2	480	50.6	28	43	-	18	[42]
*n* = 1	140	15	11	23	-	5	[42]
*n* = 1	210	65	42	83	-	62	[42]
**Stomach contents**							
*n* = 2	24	1	0.6	1	-	-	[45]
*n* = 4	130	28.8	10	29	-	6	[42]
*n* = 1	160	50	16	40	-	13	[42]
*n* = 2	140	32	12	34	-	12	[42]
*n* = 1	110	11	5	14	-	2	[42]
**Brain**							
*n* = 6	2	1	2	9	-	-	[45]
*n* = 1	420	125	51	110	-	-	[42]
**Muscle**							
*n* = 3	9	1	1	2	-	-	[45]
*n* = 7	-	361 ^3^	-	-	1144 ^3^	-	[46]
**Kidney**							
*n* = 6	16	1	2	8	-	-	[45]
**Mantak**							
*n* = 4	68.5	40.7	48.2	113	46	-	[47]
**Bowhead whale**							
**Blubber**							
*n* = 17	410	331	152	100	203	84	[49]
*n* = 5	218	4680	149	184	67	-	[43]
*n* = 3	318	-	-	23.8	-	-	[48]
**Liver**							
*n* = 23	9.1	3.7	5.4	3.1	9.5	3	[49]
**Meat**							
*n* = 4	27.2	-	-	0.6	-	-	[48]
**Mungtak**							
*n* = 7	142.6	-	-	11.6	-	-	[48]

^1^ Wet weight; ^2^ The hyphen indicates that no data are available; ^3^ in ng/g lipids. PCB, polychlorinated biphenyls; ΣDDT, sum of o,p′-DDD, p,p′-DDD, o,p′-DDE, p,p′-DDE, o,p′-DDT, and p,p′-DDT; CHLOR, chloranes; HCH, hexachlorocyclohexane.
